# Enhanced Hydrazine Electrooxidation Activities on Novel Benzofused Tricyclic Heterocyclic Derivatives

**DOI:** 10.1021/acsomega.4c04834

**Published:** 2024-09-17

**Authors:** Omruye Ozok Arıcı, Raffaella Mancuso, Bartolo Gabriele, Arif Kivrak, Hilal Kivrak

**Affiliations:** † Department of Biomedical Engineering, Faculty of Engineering and Architectural Sciences, Eskisehir Osmangazi University, 26040 Eskisehir, Turkey; ‡ Laboratory of Industrial and Synthetic Organic Chemistry (LISOC), Department of Chemistry and Chemical Technologies, University of Calabria, Via Pietro Bucci 12/C, 87036 Arcavacata di Rende (CS), Italy; § Department of Chemistry, Faculty of Sciences and Arts, Eskisehir Osmangazi University, 26040 Eskişehir, Turkey; d Department of Chemical Engineering, Faculty of Engineering and Architectural Sciences, Eskisehir Osmangazi University, 26040 Eskisehir, Turkey

## Abstract

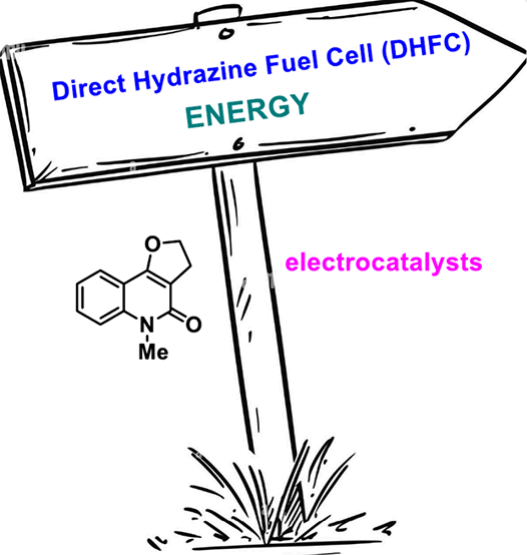

In this study, some benzofused tricyclic heterocyclic derivatives have been tested as possible new catalysts for anode hydrazine electrooxidation in a direct hydrazine fuel cell (DHFC). Electrochemical studies were carried out in solution media containing 1 M KOH and 1 M KOH + 0.5 M N_2_H_4_ using electrochemical techniques such as cyclic voltammetry (CV), electrochemical impedance spectroscopy (EIS), and chronoamperometry (CA). The CV results obtained showed that 5 catalysts promoted the generation of the best current (38.32 mA/cm^2^), and EIS results confirmed that an electrode modified with the same derivative presented the lowest charge transfer resistance. All these results proved that 5 organic-based catalysts can be used as an anode-efficient catalyst in hydrazine fuel cells.

## Introduction

Due to the nonrenewability and environmental impact associated with the use of fossil fuels, the implementation of alternative, affordable, and clean energy sources has become inevitable.^1^ In this regard, fuel cell-based technologies are destined to play a major role in the near future, as they are able to produce clean energy by converting chemical energy into electrical energy without the need for fossil fuels and without direct emission of carbon dioxide.^2^

In particular, thanks to their high cell voltage production, direct hydrazine fuel cells (DHFCs) have been attracting increasing attention from researchers, from both academia and industry.^3^ DHFCs are of particular importance, since they are characterized by a high fuel conversion rate, low cost, and good durability.^4^ Due to its low electrooxidation potential (−1.21 V) and high hydrogen concentration (12.5% by weight),^5,6^ hydrazine (N_2_H_4_) is the preferred fuel for fuel cells. The following equations could be used to explain the mechanism of the electrooxidation of N_2_H_4_ in an alkaline environment:^7^Anode Reaction:
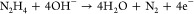
1Cathode Reaction:
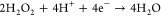
2Overall Reaction:
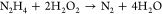
3

Clearly, no CO_2_ is produced in these reaction, which is very important, as carbon dioxide could poison the electrocatalysts necessary for promoting the electrochemical process.^6^

A variety of catalysts with high electrocatalytic activity and low cost have been developed as anode catalysts for DHFC in the literature. For instance, Ni–Co,^8^ Ni_3_S2@Ni,^9^ AgNi/MWCNT,^10^ Cu–Ni,^11^ nano-CuO/MGCE,^12^ NiMo/C,^13^ Ni–Pt/C,^14^ NPCF/Cu,^15^ CuNiCo LDH,^16^ and PtCofiber/Cu^4^ are reported for N_2_H_4_ electrooxidation reactions as anode catalysts. Table 1 displays the maximum current density peak values achieved with these metal-based catalysts.

**Table 1:** Maximum Current Density and Initial Potential Values for N_2_H_4_ Electrooxidation Reported in the Literature

Catalyst	Onset Potential (V)	Current Density (mA/cm^2^)	Preparation	Reference
AuPd NCs	-	5.28	chemical reduction	(21)
PpDP/ZnO	–0.06	7.89	dope	(22)
Wash-CNCu	0.28	1.94	dope	(23)
NiFe2O4-rGO	0.36	18.9	hydrothermal method	(24)
NSC	0.6	7.89	chemical reduction	(25)
benzo[*b*]thiophene	–0.20	4.95	organic synthesis	(20)
compound **5**	0.2	38.32	organic synthesis	this study

Due to the disadvantages of metals (such as their toxicity, high cost, and non-reusability), organic materials have recently been proposed as anode catalysts in new generation DHFC applications.^17^ In particular, a metal-free anode catalyst has been recently reported by Sharif and co-workers, and 2-isopropyl-5-methylphenyl-4-oxo-4-(5-(*p*-tolylethynyl)thiophene-2-yl butanoate organic catalyst exhibited the highest performance with a current density value of 3.66 mA cm^–2^ (17.24 mA mg^–1^).^18^ Other environmentally friendly, inexpensive organic materials have been developed in place of expensive metal catalysts (such as Pt and Au) as anode catalysts in DHFCs.^19,20^

**Scheme 1:**
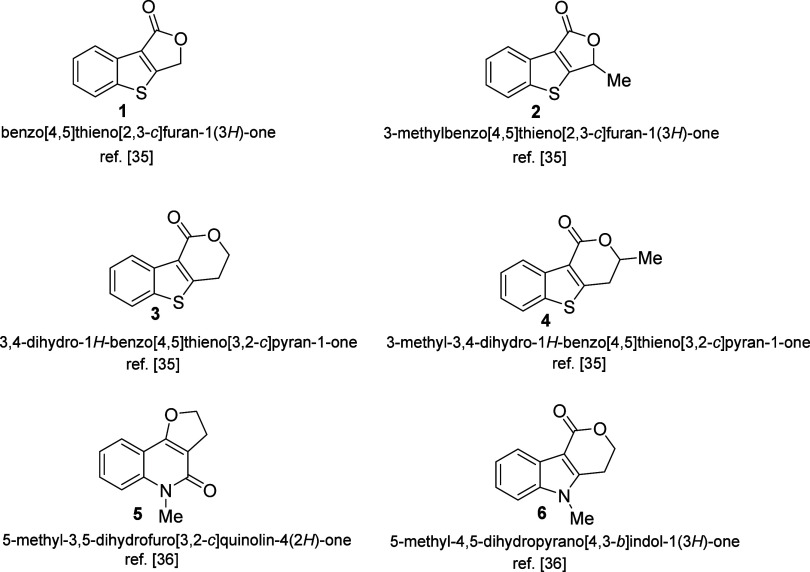
Polycyclic Heterocycles Investigated in This Study as Possible Electrocatalysts for Direct Hydrazine Fuel Cell (DHFC) Applications

Polycyclic heterocycles are a particularly important class of heterocyclic systems, as they are present as fundamental cores in natural products and in bioactive principles^26^ and can also be used for applications in materials sciences.^27^ In this study, we tested some recently synthesized polycyclic heterocycles as possible anode catalysts with high catalytic activity and long-term stability in hydrazine electrooxidation. In particular, we focused our attention on six derivatives recently reported by our research group,^28,29^ as shown in Scheme 1.

Compared to metal-based catalyst systems, organic-based anode catalysts can be easily synthesized with different kinds of derivatives, and they can be tested for finding better anode catalysts in DHFC applications. In addition, they do not exhibit any sensitivity to oxygen. Accordingly, the electrocatalytic activity of compounds **1**–**6** for the electrooxidation of N_2_H_4_ has been evaluated with the help of cyclic voltammetry (CV), chronoamperometry (CA), and electrochemical impedance spectroscopy (EIS).

## 2 Materials and Methods

### 2.1 Synthesis

Polycyclic heterocycles **1**–**4**^28^ and **5**/**6**^29^ were prepared according to the published procedures.

### 2.2 Electrochemical Studies

Electrochemical methods including CV, CA, and EIS were performed (1 M KOH + 0.5 M N_2_H_4_ solution) using a Gamry-Interface 1010 potentiostat fitted with a 3-electrode system in order to examine the hydrazine electrooxidation (HEO) activity of 1–6 organic catalysts. The working electrode consists of a 3.0 mm diameter glassy carbon disk held in a Teflon cylinder. Before the procedures, electrode surfaces were polished and immediately rinsed with tap water, distilled water, and acetone. In order to create electrodes from catalysts, 5 mg of catalyst was first homogenized in an ultrasonic bath for 10 min with 1 mL of Nafion solution (Nafion 117, Aldrich, comprising 5% Nafion). A volume of 3–5 μL of the resulting catalyst + naphthione mixture was transferred with a micropipet and applied to a 3 mm diameter glassy carbon electrode. The electrodes were then allowed to dry. The stability of the organic catalyst was evaluated using CA measurements over a 1000 s period at potentials of −0.8 V/0.6 V. The EIS was used for the investigation of electrochemical resistance at 316 kHz and 0.046 Hz to 0.005 V amplitude, with varying potentials in the range of −0.8/0.6 V.

## 3 Results and Discussion

**Figure 1:**
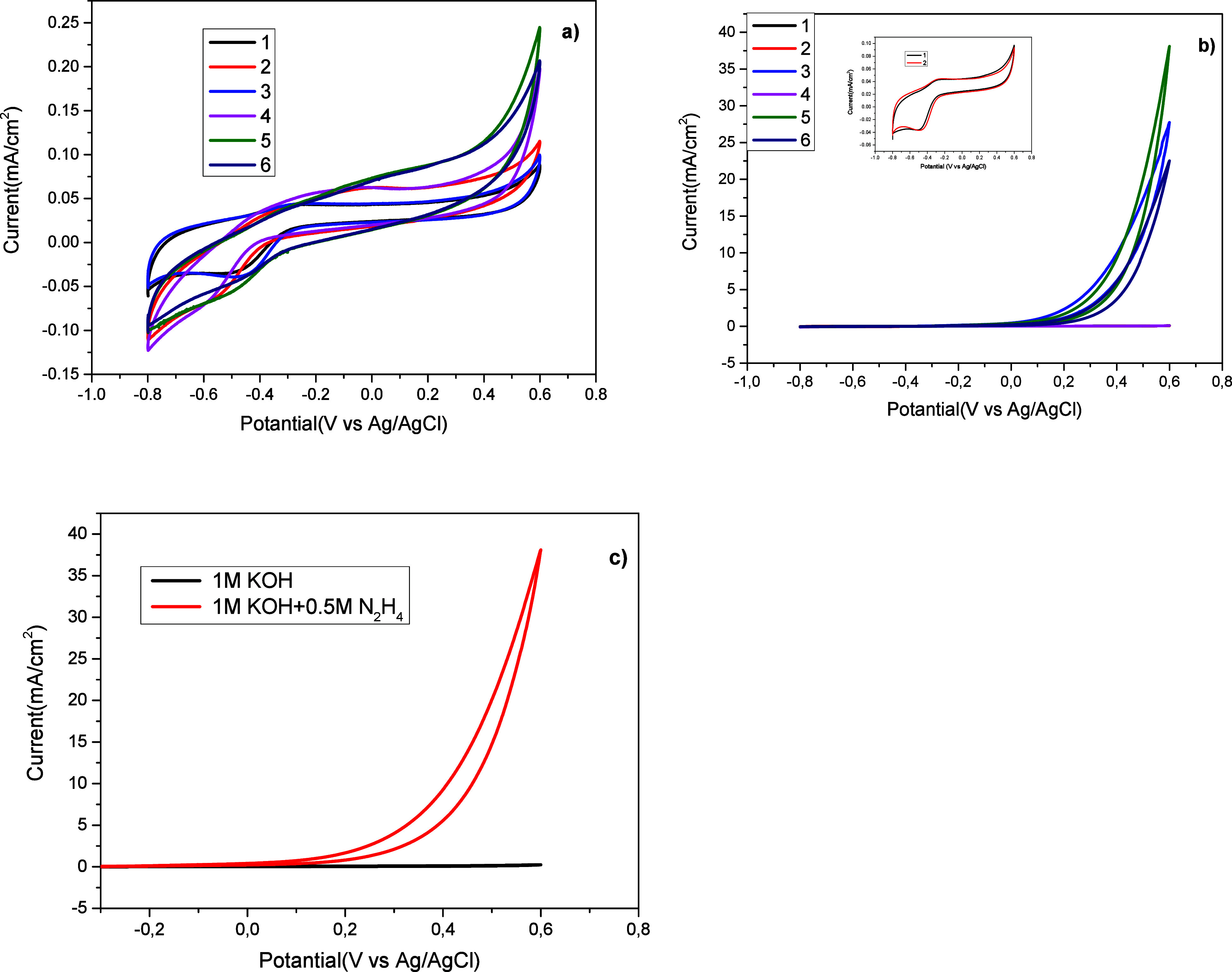
CV results of organocatalysts **1**–**6** in (a) 1 M KOH and (b) 1 M KOH + 0.5 M N_2_H_4_. (c) CV result of catalyst **5** in 1 M KOH and 1 M KOH + 0.5 M N_2_H_4_.

The HEO activity of organic-based catalysts **1**–**6** was identified by CV analysis. Hydrazine electrooxidation studies on organic-based catalysts were performed in 1 M KOH solution and 1 M KOH + 0.5 M N_2_H_4_ solution at a scan rate of 50 mV s^–1^ and −0.8 and 0.6 V potentials (Figure 1a–c). As it is known, when metal-based catalysts such as Pd, Pt, and Au are used for electrooxidation, significant oxidation peaks are observed in CV. However, these oxidation peaks cannot be observed when organic catalysts are used. The specific activity of compounds **1**–**6** for HEO obtained from CV results is given in Table 2. As can be seen in Figure 1b and Table 2, organocatalyst **5** exhibited better activity than other catalysts. The specific activity in the total current of N_2_H_4_ was calculated as 38.32 mA cm^–2^. When the electrooxidation performance was carried out in the presence of hydrazine without any organic catalyst, we did not obtain any peaks.

**Table 2:** HEO Performances of Organic-Based Catalysts

Catalyst	Onset Potential (V)	Total Current of KOH (mA/cm^–2^)	Total Current of Hydrazine(mA/cm^–2^)	Normal Current, (mA/cm^–2^)
**1**	–0.4	0.08	0.17	0.09
**2**	–0.4	0.11	0.20	0.09
**3**	0.1	0.09	27.60	27.51
**4**	–0.4	0.19	0.29	0.10
**5**	**0.2**	**0.24**	**38.32**	**38.08**
**6**	0.1	0.20	22.53	22.33

**Figure 2:**
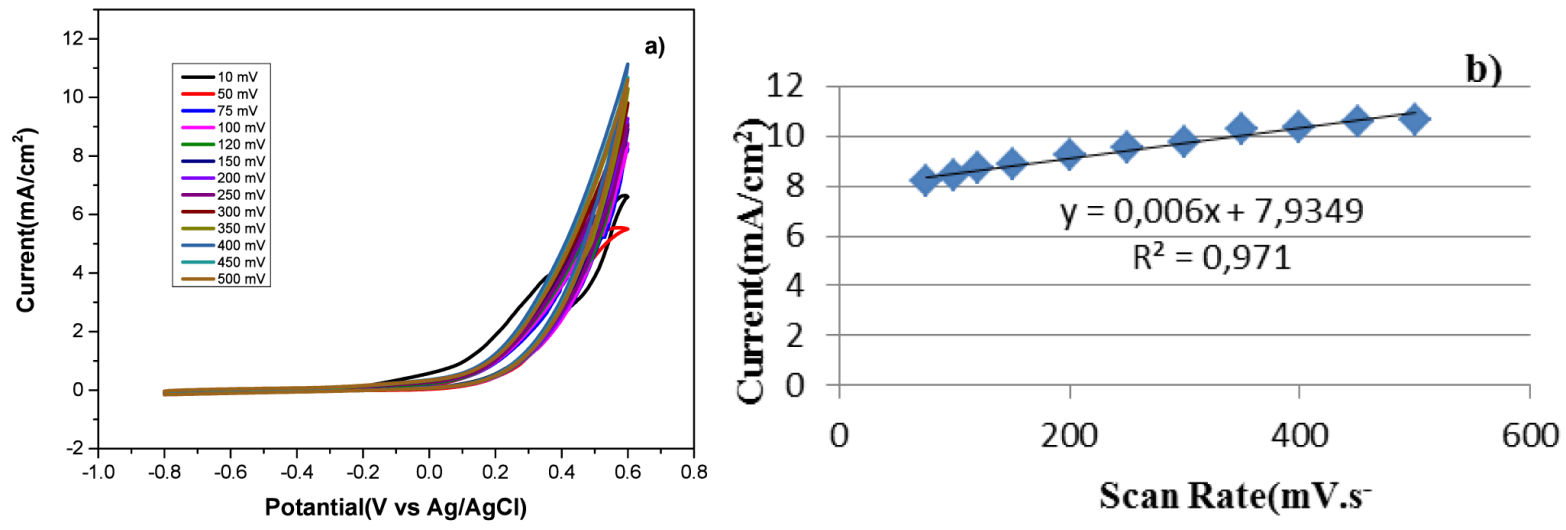
(a) CV analysis of organocatalyst **5** at different scan rates (10–500 mV s^–1^). (b) Linear regression of peak currents vs the square root of the scan rates.

Figure 2a,b display the analysis of the hybrid organocatalyst **5**, which was obtained at various scan rates (10 and 500 mV s^–1^) in 1 M KOH + 0.5 M N_2_H_4_. Plotting the current value against the square root of the scan rate shows that it increases and changes linearly with the scan rate (see the graph’s inset). This is evidence of a diffusion-controlled reaction for the HEO.

**Figure 3:**
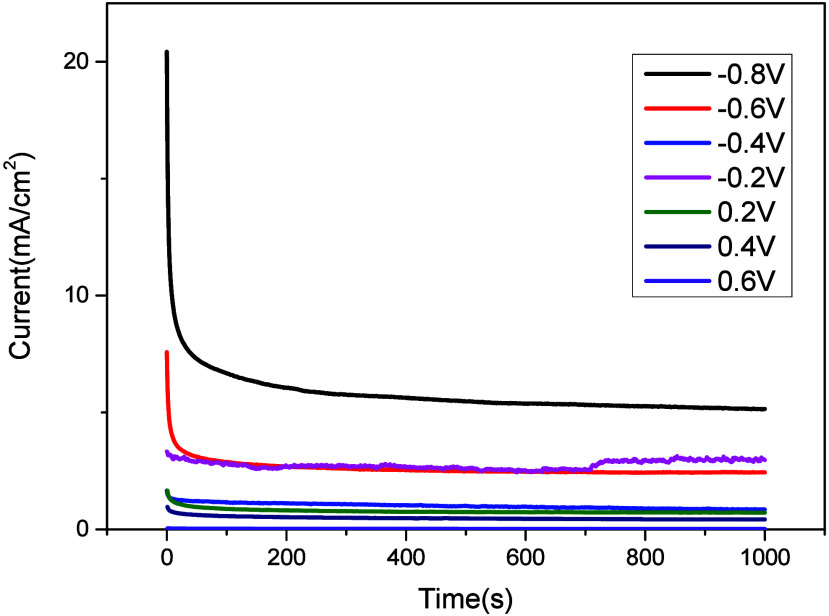
CA analysis of organocatalyst **5**.

The stability of organocatalyst **5** was investigated using CA measurements. The CA curves of organocatalyst **5** at various potentials are shown in Figure 3. CA analysis proved that the best resistance and good stability were obtained at a potential of less than 0.8 V.

**Figure 4:**
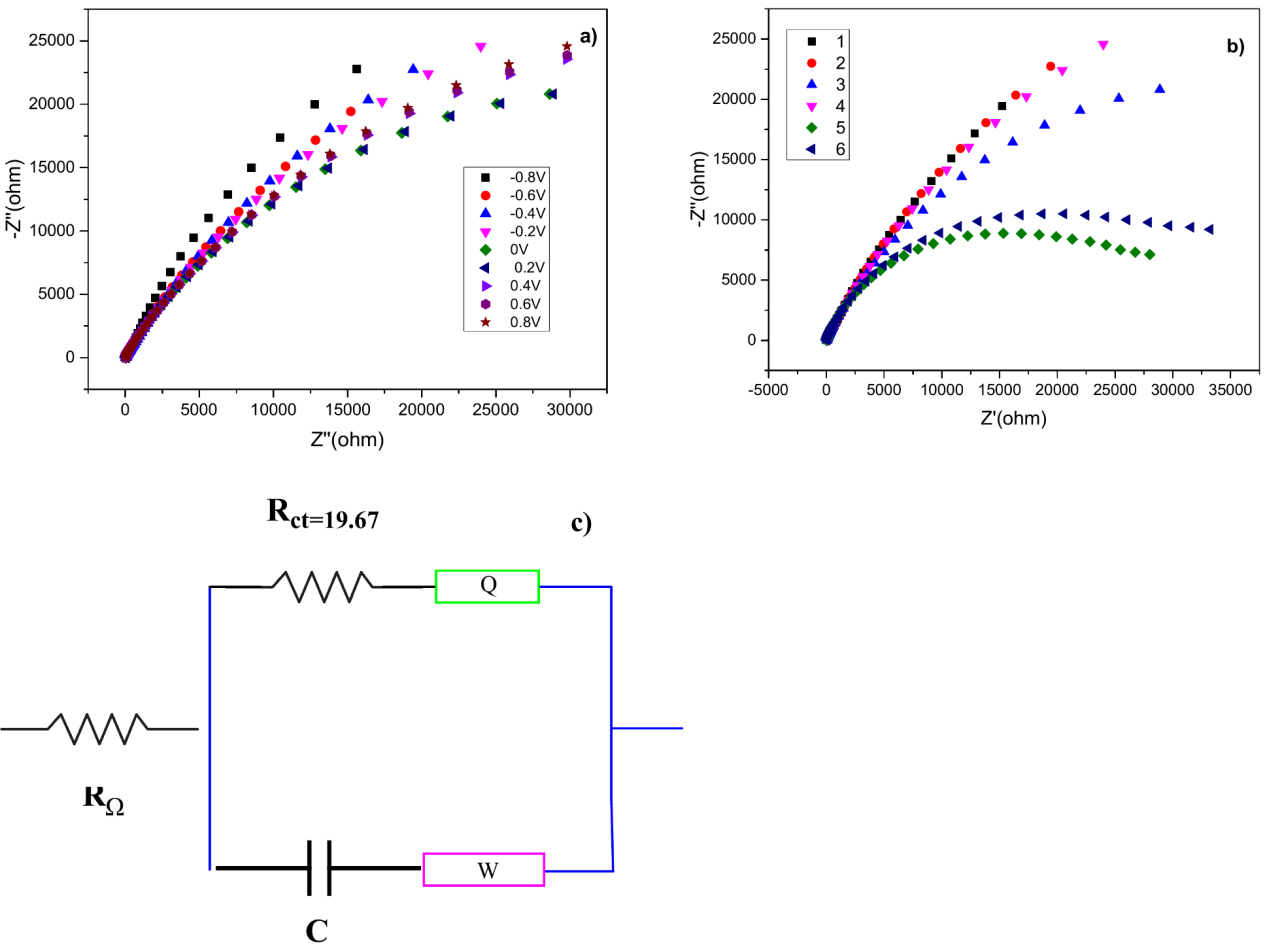
Nyquist plots obtained (a) at different potentials of organocatalyst **5** and (b) compared to all other organocatalysts at 0.2 V in 1 M KOH + 0.5 M N_2_H_4_; (c) *R*_ct_ value.

The electrocatalytic resistance of organocatalyst **5** was investigated by using the Nyquist plots from the EIS, as shown in Figure 4a,b. EIS measurements are often used to calculate electrocatalytic resistance, with the diameter of the circle decreasing as resistance increases.^30^Figure 4a displays the Nyquist plots of the hybrid **5** organic catalyst achieved at different potentials (−0.8, −0.6, −0.4, −0.2, 0.0, 0.2, 0.4, 0.6, and 0.8 V). As seen in Figure 4a, the Nyquist graph showed that the highest electrochemical resistance was obtained when the organic catalyst **5** was taken at 0.2 V. Figure 4b shows the Nyquist plots of organocatalysts **1**–**6** at a potential of 0.2 V. Organocatalyst **5** gives a smaller *R*_ct_ value than other catalysts. In DHFC applications, EIS studies also proved that **5** has the best catalytic activity, lowest resistance, and high stability.

In Figure 4c, the charge transfer resistance (*R*_ct_) values of the **5**-based electrode in 1 M KOH + 0.5 M N_2_H_4_, −0.8 V (39.89 Ω) > −0.6 V (39.22 Ω) > −0.4 V (38.82 Ω) > −0.2 V (38.75 Ω), > 0 V (38.68 Ω), > 0.2 V (19.67 Ω), > 0.4 V (37.07 Ω), and 0.6 V (36.08 Ω), were found from the different voltage equivalent circuit model. In comparison to other voltages, the electrode of organocatalyst **5** exhibits the maximum carrier transfer performance due to its lowest semicircular shape and *R*_ct_ of 0.2 V (Table 3).

**Table 3:** Charge Transfer Resistance (*R*_ct_) Values of the C1 Electrode at Different Voltages

Electrode	–0.8 V	–0.6 V	–0.4 V	–0.2 V	0 V	0.2 V	0.4 V	0.6 V
**5**	39.89	39.22	38.82	38.75	38.68	19.67	37.07	36.08

## Conclusions

In conclusion, in this study, we tested 6 different benzofused tricyclic heterocyclic derivatives as possible new catalysts for anode hydrazine electrooxidation in a DHFC. The CV results obtained showed that 5 catalysts promoted the generation of the best current (38.32 mA/cm^2^), and EIS results confirmed that the electrode modified with the same derivative presented the lowest charge transfer resistance. As a result, this study improved the possibility that compound **5** could be used in hydrazine fuel cells as an anode catalyst without needing any metal.
